# Correction: Onodera et al. Prognostic Factors and Talaporfin Sodium Concentration in Photodynamic Therapy for Recurrent Grade 4 Glioma. *Pharmaceuticals* 2025, *18*, 583

**DOI:** 10.3390/ph18101586

**Published:** 2025-10-21

**Authors:** Mikoto Onodera, Shuji Kitahara, Yasuto Sato, Takakazu Kawamata, Yoshihiro Muragaki, Ken Masamune

**Affiliations:** 1 Faculty of Advanced Techno-Surgery (FATS), Institute of Advanced Biomedical Engineering and Science, Tokyo Women’s Medical University, 8-1 Kawada-Cho, Shinjuku, Tokyo 162-8666, Japan; onodera.mikoto@twmu.ac.jp (M.O.); muragaki@people.kobe-u.ac.jp (Y.M.); masamune.ken@twmu.ac.jp (K.M.); 2 Department of Pharmacy, Tokyo Women’s Medical University, 8-1 Kawada-Cho, Shinjuku, Tokyo 162-8666, Japan; 3 Department of Hygiene and Public Health, Tokyo Women’s Medical University, 8-1 Kawada-Cho, Shinjuku, Tokyo 162-8666, Japan; sato.yasuto@twmu.ac.jp; 4 Graduate School of Public Health, Shizuoka Graduate University of Public Health, 4-27-2 kita Ando, Aoi-ku, Shizuoka, Shizuoka 420-0881, Japan; 5 Department of Neurosurgery, Tokyo Women’s Medical University, 8-1 Kawada-Cho, Shinjuku, Tokyo 162-8666, Japan; tkawamata@twmu.ac.jp; 6 Center for Advanced Medical Engineering Research and Development, Kobe University, 1-5-1 Minatojima Minamimachi, Chuo-Ku, Kobe, Hyogo 650-0047, Japan

## Error in Table

In the original publication [1], there were mistakes in a part of Table 2, Section 2.5, and the label of Table 3. In Table 2, errors present in the data shown under “Recurrence” were identical to those under “Newly Diagnosed”. The corrected Table 2 appears below.

The values in Section 2.5 of the text did not match those in Table 3. We have replaced the first paragraph in Section 2.5 with the paragraph below, in which the numerical values have been corrected, as shown in Table 3. In addition, the label of the right-hand group in Table 3 was incorrect; the correct label is “Recurrence.” The corrected Table 3 appears below. The authors state that the scientific conclusions are unaffected. This correction was approved by the Academic Editor. The original publication has also been updated.

### 2.5. Analysis of Clinical Factors in the High vs. Low Intratumoral TS Concentration Groups

Among the 82 patients for whom intratumoral TS concentration was measured, the newly diagnosed group consisted of 41 patients (high concentration, 51.2%; low concentration, 48.8%) with a mean age of 60.7 years (SD: 12.2 years) and 61.3 years (SD: 11.9 years), respectively. 

Similarly, the recurrent group included 41 patients (high concentration, 51.2%; low concentration, 48.8%) with a mean age of 47.6 years (SD: 14.2 years) and 53.6 years (SD: 10.5 years), respectively. 

No significant differences in age or sex were observed between the high- and low-concentration groups (age, Newly Diagnosed *p* = 0.884 and Recurrence *p* = 0.5843, and sex, Newly Diagnosed *p* = 0.837 and Recurrence *p* = 0.5843, respectively). Additionally, no significant differences were found in tumor location, genetic mutations, benzodiazepine use, or smoking history, suggesting that no specific factors were associated with TS concentrations (Table 3).

**Table 2:** Analysis of risk factors among the two groups of patients who underwent PDT between January 2017 and March 2024: newly diagnosed (*n* = 74) and recurrent (*n* = 97).

	Newly Diagnosed				Recurrence			
	Hazard Ratio (95% CI)	*p*-Value	Hazard Ratio (95% CI)	*p*-Value	Hazard Ratio (95% CI)	*p*-Value	Hazard Ratio (95% CI)	*p*-Value
Age	1.02 (0.99–1.04)	0.26	1.02 (0.99–1.05)	0.27	1.01 (0.99–1.03)	0.22	2.03 (0.81–5.93)	0.50
Male vs. female sex	1.35 (0.62–2.94)	0.44			1.08 (0.62–1.88)	0.45		
KPS	0.98 (0.95–1.00)	0.097	0.96 (0.93–0.99)	0.01	0.09 (0.02–0.33)	0.0003	0.96 (0.95–0.99)	0.0008
Alb	1.51 (0.51–4.48)	0.45	3.68 (1.05–13.76)	0.047	2.22 (0.88–5.84)	0.090	2.13 (0.81–5.93)	0.13
Total cholesterol	1.00 (0.98–1.01)	0.59			1.00 (0.99–1.01)	0.40		
Benzodiazepine	2.36 (0.81–6.88)	0.15	3.06 (0.96–9.76)	0.059	0.77 (0.34–1.72)	0.52	0.77 (0.34–1.74)	0.52
Smoking	1.57 (0.70–3.53)	0.29	1.58 (0.67–3.73)	0.3	1.07 (0.64–1.79)	0.80	1.21 (0.67–2.19)	0.53
Localization								
Temporal vs. not temporal	0.50 (0.22–1.11)	0.079			1.00 (0.56–1.78)	0.99		
Frontal vs. not frontal	1.14 (0.51–2.56)	0.75			0.72 (0.43–1.20)	0.20		
Parietal vs. not parietal	1.32 (0.53–3.31)	0.56			1.36 (0.72–2.53)	0.35		

**Table 3:** Comparison between the newly diagnosed and recurrent groups based on median tissue TS concentration.

	Newly Diagnosed			Recurrence		
	Mean TS Concentration ≤0.16333	Mean TS Concentration >0.16333		Mean TS Concentration ≤0.0883	Mean TS Concentration >0.0883	
	*n* = 21 51.2 (%)	*n* = 20 48.8 (%)	*p*-Value	*n* = 21 51.2 (%)	*n* = 20 48.8 (%)	*p*-Value
Male vs. female	13 (31.7%)/8 (19.5%)	13 (31.7%)/7 (17.1%)	0.837	13 (31.7%)/8 (19.5%)	14 (34.2%)/6 (14.6%)	0.5843
Age, mean ± SD	60.7 ± 12.2	61.3 ± 11.9	0.884	47.6 ± 14.2	53.6 ± 10.5	0.5843
Left vs. right (excluded bilateral case)	11 (31.4%)/7 (20.0%)	6 (17.1%)/11 (31.4%)	0.1245	10 (26.3%)/9 (23.7%)	6 (15.8%)/13 (34.2%)	0.1869
Pathological diagnosis						
Mean MIB-1 ± SD	28.9 ± 11.9	22.1 ± 11.5	0.0607	21.3 ± 15.4	22.0 ± 15.5	0.8886
ATRX intact vs. loss	20 (51.2%)/0 (0%)	18 (46.2%)/1 (2.6%)	0.226	12 (30.8%)/7 (18.0%)	16 (39.0%)/4 (9.7%)	0.2407
P53 (+) vs. (−)	4 (9.76%)/17 (41.5%)	6 (14.6%)/14 (34.2%)	0.4134	6 (15.0%)/14 (35.0%)	7 (17.0%)/13 (31.7%)	0.7356
IDH (+) vs. (−)	0 (0%)/21 (51.2%)	1 (2.4%)/19 (46.3%)	0.226	5 (12.5%)/15 (37.5%)	4 (9.7%)/16 (39.0%)	0.7047
Part						
Temporal vs. not temporal	7 (17.1%)/14 (34.2%)	9 (22.0%)/11 (26.8%)	0.4435	8 (19.5%)/13 (31.7%)	5 (12.2%)/15 (36.6%)	0.366
Frontal vs. not frontal	11 (26.8%)/10 (24.4%)	8 (19.5%)/12 (29.3%)	0.4261	7 (17.1%)/14 (34.2%)	8 (19.5%)/12 (29.3%)	0.6577
Parietal vs. not parietal	2 (4.9%)/19 (46.3%)	2 (4.9%)/18 (43.9%)	0.959	5 (12.2%)/16 (39.0%)	5 (12.2%)/15 (36.6%)	0.9293
